# Efficient callus formation and plant regeneration are heritable characters in sugar beet (*Beta vulgaris* L.)

**DOI:** 10.1186/s41065-016-0015-z

**Published:** 2016-11-15

**Authors:** Hiroyo Kagami, Kazunori Taguchi, Takumi Arakawa, Yosuke Kuroda, Hideto Tamagake, Tomohiko Kubo

**Affiliations:** 1 Research Faculty of Agriculture, Hokkaido University, Sapporo, 060-8589 Japan; 2 Hokkaido Agricultural Research Center, National Agriculture and Food Research Organization, Memuro, 082-0081 Japan; 3 Central Agricultural Experimental Station, Agriculture Research Department, Hokkaido Research Organization, Takikawa, 073-0013 Japan

**Keywords:** Dedifferentiation, DNA marker, F_1_ hybrid, in vitro culture, Somatic embryo

## Abstract

**Background:**

Obtaining dedifferentiated cells (callus) that can regenerate into whole plants is not always feasible for many plant species. Sugar beet is known to be recalcitrant for dedifferentiation and plant regeneration. These difficulties were major obstacles for obtaining transgenic sugar beets through an *Agrobacterium*-mediated transformation procedure. The sugar beet line ‘NK-219mm-O’ is an exceptional line that forms callus efficiently and is easy to regenerate, but the inheritance of these characters was unknown. Another concern was whether these characters could coexist with an annual habitat that makes it possible to breed short life-cycle sugar beet suitable for molecular genetic analysis.

**Findings:**

Five sugar beet lines including NK-219mm-O were crossed with each other and subjected to in vitro culture to form callus. F_1_s with a NK-219mm-O background generally formed callus efficiently compared to the others, indicating that efficient callus formation is heritable. The regeneration potential was examined based on the phenotypes of calli after placement on regeneration medium. Five phenotypes were observed, of which two phenotypes regenerated shoots or somatic embryo-like structures. Vascular differentiation was evident in regenerable calli, whereas non-regenerable calli lacked normally developed vascular tissues. In a half-diallel cross, the callus-formation efficiency and the regeneration potential of reciprocal F_1_s progeny having a NK-219mm-O background were high. Finally, we crossed NK-219mm-O with an annual line that had a poor in vitro performance. The callus-formation efficiency and the regeneration potential of reciprocal F_1_ were high. The regenerated plants showed an annual habitat.

**Conclusions:**

Efficient callus formation and the high plant regeneration potential of NK-219mm-O were inherited and expressed in the F_1_. The annual habitat does not impair these high in vitro performances.

**Electronic supplementary material:**

The online version of this article (doi:10.1186/s41065-016-0015-z) contains supplementary material, which is available to authorized users.

## Findings

Transgenic plants play pivotal roles in molecular genetic analysis and crop biotechnology. To obtain transgenic plants, *Agrobacterium*-mediated transformation techniques have been devised for many crops [1]. In some cases, explants are dedifferentiated in vitro to obtain callus that is subsequently infected with *Agrobacterium* harboring a recombinant Ti plasmid that will be inserted into plant chromosomes. The transformed cells are subsequently induced to regenerate whole plants.

Sugar beet (*Beta vulgaris* L.) is generally recalcitrant for dedifferentiation and plant regeneration, although cultivars and breeding lines show different responses to in vitro culture. This finding suggests that dedifferentiation and plant regeneration may be heritable characters and the genes involved in these characters may be scarce in sugar beet populations. In fact, Tomita et al. [2] investigated 61 sugar beet lines regarding their responses to in vitro culture and found variation in the frequencies of callus formation and somatic-embryo formation; however, the inheritance of these characters was not examined.

The sugar beet line ‘NK-219mm-O’, developed by the Hokkaido Agricultural Research Center (HARC), Japan, is an exceptional genotype that forms callus and regenerates plants very efficiently. These attributes enabled us to develop an *Agrobacterium*-mediated transformation system for sugar beet [3, 4]. Using this system, molecular analyses of several genes have been completed [5–7]. On the other hand, the shortcomings of NK-219mm-O include its vernalization requirement, i.e. the line must experience a certain duration of low temperatures as a prerequisite for flowering. Examining, for example, flower phenotypes in transgenic sugar beet takes a long time due to the vernalization requirement, which is a rate limiting step for the molecular genetic analysis of sugar beets.

The vernalization requirement in sugar beet is genetically conditioned by a recessive allele of the *b* gene (bolting) [8]. The dominant allele *B* makes vernalization unnecessary for flowering. The detailed molecular organization of the *B* locus was elucidated, and a DNA marker discriminating *b* from *B* was reported [8]. If the *B* gene does not interfere with genes governing callus formation and plant regeneration in NK-219mm-O, it may be possible to breed an annual sugar beet that is suitable for molecular genetic analysis.

First, we wished to determine the inheritance of callus formation and plant regeneration of NK-219mm-O. The parental sugar beet lines were five inbred lines, NK-219mm-O, NK-195mm-O, NK-235mm-O, NK-239mm-O, and NK-294mm-O, all of which were developed by HARC for hybrid breeding using cytoplasmic male sterility (CMS), which is genetically conditioned by male sterility-inducing cytoplasm (S) and a recessive allele of *rf* (restorer of fertility). A CMS line is produced by repeated backcrossing (more than four times) of a CMS line with a pollen parental line with the *rfrf* genotype but with normal fertile cytoplasm (N) to secure pollen production. This pollen parental line is called a maintainer or an O-type in sugar beet terminology. As such, a CMS line and its cognate O-type are near-identical in nuclear genotypes but differ in their cytoplasms. CMS and O-type lines are discriminated by suffixes ‘-CMS’ and ‘-O’, respectively. A shared number in the prefix (e.g. ‘219’) indicates that the two lines have nearly identical nuclear genotypes.

The five parental lines were crossed to obtain ten F_1_ populations, NK-195mm-CMS x NK-219mm-O, NK-195mm-CMS x NK-235mm-O, NK-195mm-CMS x NK-239mm-O, NK-195mm-CMS x NK-294mm-O, NK-219mm-CMS x NK-235mm-O, NK-219mm-CMS x NK-294mm-O, NK-219mm-CMS x NK-239mm-O, NK-235mm-CMS x NK-239mm-O, NK-235mm-CMS x NK-294mm-O, and NK-239mm-CMS x NK-294mm-O (in this report, crosses are denoted as seed parent x pollen parent). We investigated the frequency of callus formation in these F_1_ populations and five parental lines. As detailed in [3, 4], surface-sterilized seeds were sown on solid medium in vitro, and plantlets were grown. We removed two leaves from each plantlet, cut each of them into six pieces, and placed the explants on callus-inducing medium. We defined callus as apparently dedifferentiated cell clumps that were friable, white or pale yellow in color, and proliferated after detachment from the explants. The presence or absence of callus was monitored for twelve weeks. Of the two leaves, the better value was considered as the plant's phenotype for callus formation. The ratio of the number of callus-forming explants to the total number of explants was calculated for each F_1_ or parental line, from which fourteen to twenty plantlets were examined. A second replicate (another two leaves from each plantlets) was examined similarly (see data set in Additional file 1). We summarize these data in Table 1 and Additional file 2: Figure S1. The frequencies of callus formation for five F_1_ populations involving NK-219mm-O or NK-219mm-CMS were high (>0.92), a result that is comparable to that of NK-219mm-O, with the exception of NK-219mm-CMS x NK-294mm-O that had a lower frequency (0.754). None of the other F_1_ populations nor the parental lines exceeded a frequency of callus formation >0.9 except NK-239mm-CMS x NK-294mm-O (0.927). The results of our quantitative genetic analyses suggested it unlikely that a complex genetic interaction is involved in callus formation (Additional file 3: Tables S1 and S2, and Additional file 2: Figure S2). Taken together, these results indicate that efficient callus formation of NK-219mm-O is a heritable character. Note that the general combining ability (GCA) and specific combining ability (SCA) are significant (Additional file 3: Table S3), suggesting that the F_1_ does not always express callus formation as efficiently as NK-219mm-O (e.g. NK-219mm-CMS x NK-294mm-O). Heritability in a broad sense and a narrow sense is high (Additional file 3: Table S4).

**Table 1:** Half-diallel table of *in vitro*-culture response in F_1_ populations and their parental lines^a^

	NK-195mm	NK-219mm	NK-235mm	NK-239mm	NK-294mm
a) Frequencies of callus formation (%; mean ± SD)					
NK-195mm	0.08 ± 0.15				
NK-219mm	0.93 ± 0.13	0.99 ± 0.04			
NK-235mm	0.38 ± 0.40	0.97 ± 0.12	0.51 ± 0.49		
NK-239mm	0.28 ± 0.32	0.75 ± 0.27	0.50 ± 0.43	0.00 ± 0.00	
NK-294mm	0.35 ± 0.32	0.94 ± 0.11	0.79 ± 0.17	0.93 ± 0.12	0.40 ± 0.24
b) Plant regeneration scores (mean ± SD)					
NK-195mm	125.00 ± 75.00				
NK-219mm	139.39 ± 57.63	174.47 ± 26.27			
NK-235mm	11.91 ± 15.96	100.86 ± 34.78	1.52 ± 5.03		
NK-239mm	91.13 ± 76.57	41.14 ± 44.30	47.37 ± 45.90	-	
NK-294mm	52.74 ± 62.84	113.60 ± 34.91	7.30 ± 12.16	15.52 ± 13.00	6.31 ± 12.08

^a^Suffixes are omitted. For cross combinations, see Findings

**Fig. 1:**
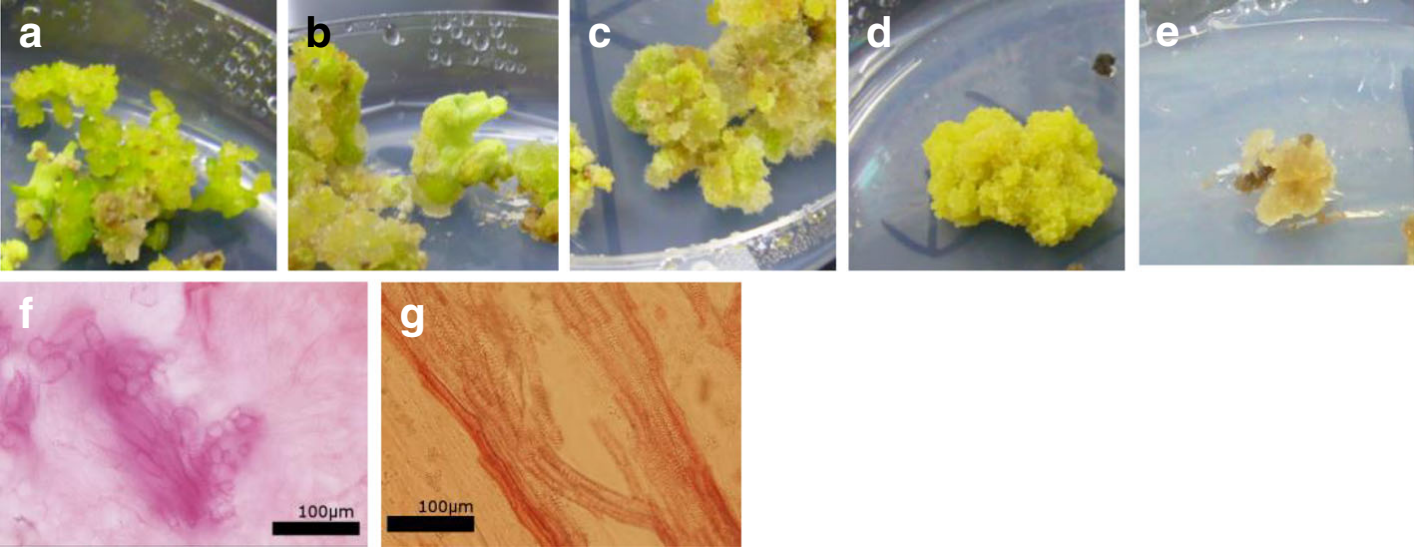
Morphology of calli on regeneration medium. Photographs **a** to **e** correspond to typical morphologies of calli Types-A to E, respectively. Photographs **f** and **g** are sections of green objects found on Type-C callus and adventitious shoots on Type-A callus, respectively. Objects were embedded in 5 % agar and sectioned into 50-μm slices using a Microslicer DTK-1000 (Dosaka EM, Kyoto, Japan). Before making microscopic observations (Olympus BX50 equipped with Olympus DP70, Olympus, Tokyo, Japan), the sections were stained with safranin

**Fig. 2:**
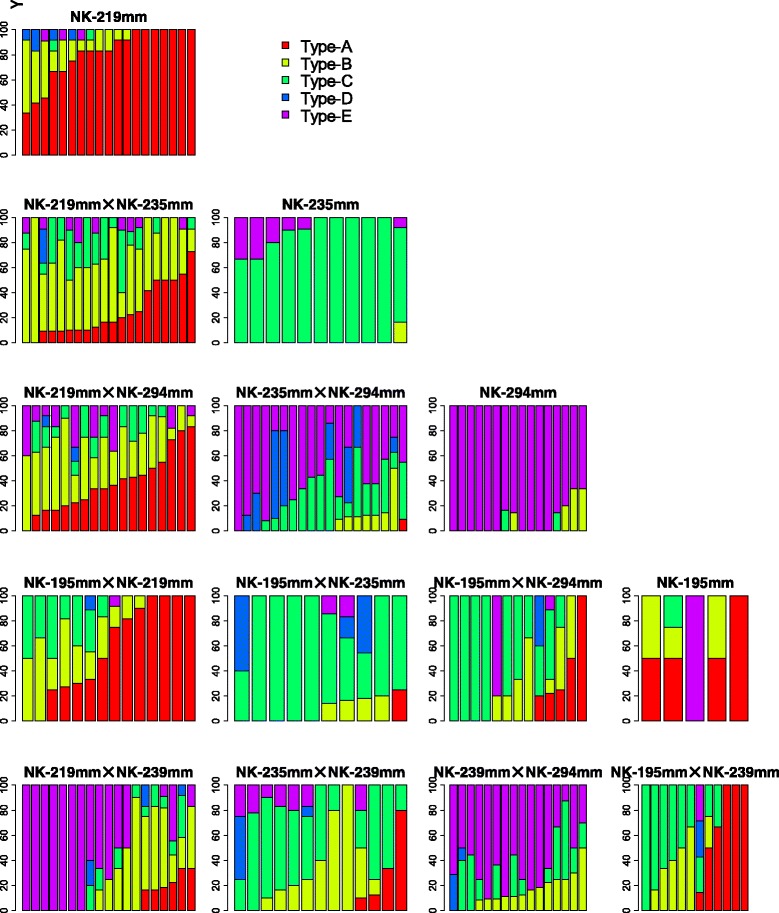
Summary of phenotypes of the parental lines and their F_1_ progeny. Suffixes (‘-O’ or ‘-CMS’) are omitted for the names of lines. For cross combinations, see Findings. NK-239mm-O was excluded because no callus was obtained from this line. Each bar represents a single plant; Y-axes are the percentage of explants having each callus phenotype. The five different phenotypes are indicated by bars of different colors: *red* (Type-A), *yellow* (Type-B), *green* (Type-C), *blue* (Type-D), and *purple* (Type-E). Types A and B can regenerate plants. Note that only five plants were examined for NK-195mm-O because the frequency of callus formation in NK-195mm-O was very low (see Table 1)

Next, we examined whether the obtained calli have the potential to differentiate into shoots. The calli of the F_1_ populations and the parental lines that were obtained in the dedifferentiation analysis were placed onto regeneration medium (see [3]) and grown for four weeks. Because there were no NK-239mm-O explants that generated callus (Table 1), this line was excluded from the regeneration analysis. Four weeks after transfer to the regeneration medium, calli had one of five of the following morphologies (see also Fig. 1): Type-A, many somatic embryo-like objects or adventitious shoots were evident; Type-B, very few somatic embryo-like objects or adventitious shoots were seen; Type-C, green objects were present that were distinct from dedifferentiated cells, somatic embryo-like objects and adventitious shoots; Type-D, calli became green but were morphologically unchanged; and Type-E, no change in color or morphology. Plantlets were obtained from somatic embryo-like objects and adventitious shoots of Types-A and B but not from the Type-C objects nor from Types-D or E calli. Sections of the green objects recovered from Type-C callus were observed by light microscopy and had irregularly arranged xylem elements; whereas, normal xylem structures were present in the adventitious shoots formed on Type-A callus (see photographs f and g in Fig. 1). Therefore, Type-C callus is distinct from Types-A and B in terms of its regeneration potential. The phenotypes of calli from each genotype were characterized by calculating the percentages of each of the five phenotypes represented (Fig. 2). Phenotypes of F_1_ plants appeared to be affected by their parental genotypes. For example, the NK-219mm-O genotype tended to have more Type-A or Type-B calli, whereas the NK-294mm-O genotype tended to have more Type-E calli.

To quantify the regeneration potential, we gave phenotypic values: 2 for Type-A calli, 1 for Type-B, and 0 for Types-C, D, and E. Using these phenotypic values, a plant's regeneration score was calculated by the following equation:

$$ Regeneration\; score=\frac{2\times {N}_A+1\times {N}_B}{N_{Total}}\kern0.62em \times \kern0.62em 100 $$

in which N_A_ is number of callus clumps exhibiting the Type-A phenotype, N_B_ is number of callus clumps exhibiting the Type-B phenotype, and N_Total_ is total number of callus clumps placed on the regeneration medium. In this equation, the maximum value for the regeneration score is 200. In Table 1, the mean values of the plants' regeneration scores in the F_1_ populations and parental lines are shown. The regeneration scores exceeded 100 in NK-195mm-O, NK-219mm-O, and F_1_ populations involving NK-219mm-O or NK-219mm-CMS with the exception of NK-219mm-CMS x NK-294mm-O (Table 1). Our quantitative genetic analyses suggested it unlikely that a complex genetic interaction is involved in the regeneration potential (Additional file 3: Tables S5 and S6, and Additional file 2: Figure S3). Because of the significance for both the GCA and the SCA (Additional file 3: Table S7), efficient plant regeneration may not always be expressed in the F_1_, as exemplified in NK-219mm-CMS x NK-294mm-O. Altogether, our results indicate that efficient plant regeneration of NK-219mm-O is a heritable character. A correlation between the frequencies of callus formation and the plant regeneration scores was unlikely considering that the correlation coefficient between these two values was insignificant (r = 0.278). Nevertheless, since both callus formation and plant regeneration of NK-219mm-O were highly heritability in a broad and narrow sense (Additional file 3: Tables S4 and S8), it is possible to select a genotype that is superior in terms of tissue culture response.

We next examined the effect of the *B* gene on in vitro culture. TA-33BB-CMS and TA-33BB-O are annual sugar beet lines developed at the HARC. Pin et al. [8] isolated the *B* gene through map-based cloning and showed that it encodes a pseudo-response regulator protein named BvBTC1. We determined part of the nucleotide sequence of *BvBTC1* cDNA from TA-33BB-O, and the data indicated that the annual habitat of TA-33BB-O is conditioned by the dominant *B* allele (Additional file 2: Figure S4).

We also examined callus formation and the regeneration potential of TA-33BB-O, TA-33BB-CMS, NK-219mm-O x TA-33BB-O, and TA-33BB-CMS x NK-219mm-O. Frequencies of callus formation were 0.1-0.11 for TA-33BB-O and TA-33BB-CMS, and 0.99-1.0 for the two F_1_ populations (Additional file 2: Figure S5 and Additional file 3: Table S9). Plant regeneration scores were 77-78 for TA-33BB-O and TA-33BB-CMS, and 182-200 for the two F_1_ populations (Additional file 3: Table S9). We obtained regenerated plants from calli of the two F_1_ populations (see Additional file 2: Figure S6). The regenerated plants flowered under 24-h day conditions without vernalization. These F_1_ populations can be propagated as somatic clones by in vitro culture and used for transgenic analyses.

## Additional files

Additional file 1: Dataset. (XLSX 37 kb)
Additional file 2: Figure S1. Distribution of callus formation frequencies in five sugar beet lines and ten F_1_ populations. **Figure S2.** A (Vr, Wr) plot for callus formation. **Figure S3.** A (Vr, Wr) plot of the regeneration scores. **Figure S4.** Nucleotide sequence alignment of a portion of *BvBTC1* from NK-219mm-O and TA-33BB-O. **Figure S5.** The distribution of callus-formation frequencies in TA-33BB-O, TA-33BB-CMS, NK-219mm-O, NK-219mm-O x TA-33BB-O, and TA-33BB-CMS x NK-219mm-O. **Figure S6.** Summary of calli phenotypes from TA-33BB-O, TA33BB-CMS, NK-219mm-O, NK-219mm-O x TA-33BB-O, and TA-33BB-CMS x NK-219mm-O. (PDF 554 kb)
Additional file 3: Table S1. Absence of epistasis in callus formation, two-way ANOVA of callus formation frequencies. **Table S2.** Absence of epistasis in callus formation, two-way ANOVA of the unity of W_r_-V_r_. **Table S3.** Combining ability and heritability of callus formation, statistics of combining ability in callus formation. **Table S4.** Combining ability and heritability of callus formation, statistics of genetic variance in callus formation. **Table S5.** Absence of epistasis in plant regeneration, two-way ANOVA of the regeneration score. **Table S6.** Absence of epistasis in plant regeneration, two-way ANOVA of the unity of W_r_-V_r_. **Table S7.** Combining ability and heritability of plant regeneration, statistics of combining ability in plant regeneration. **Table S8.** Combining ability and heritability of plant regeneration, statistics of combining ability in plant regeneration. **Table S9.** *In vitro* performances of TA-33BB-O, TA-33BB-CMS, NK-219mm-O, and their F_1_ populations. (PDF 110 kb)

## Abbreviations

*B* (*b*): Bolting
CMS: Cytoplasmic male sterility
HARC: Hokkaido Agricultural Research Center
N: Normal fertile cytoplasm
*rf*: Restorer of fertility
S: Male sterility-inducing cytoplasm
