# Supplementary material for: Efficient callus formation and plant regeneration are heritable characters in sugar beet (Beta vulgaris L.)
Source: Hereditas. 2016 Nov 15;153:12. doi: 10.1186/s41065-016-0015-z (PMC5226093; doi:10.1186/s41065-016-0015-z)
Supplement: Additional file 2: Figure S1. — Distribution of callus formation frequencies in five sugar beet lines and ten F1 populations. Figure S2. A (Vr, Wr) plot for callus formation. Figure S3. A (Vr, Wr) plot of the regeneration scores. Figure S4. Nucleotide sequence alignment of a portion of BvBTC1 from NK-219mm-O and TA-33BB-O. Figure S5. The distribution of callus-formation frequencies in TA-33BB-O, TA-33BB-CMS, NK-219mm-O, NK-219mm-O x TA-33BB-O, and TA-33BB-CMS x NK-219mm-O. Figure S6. Summary of calli phenotypes from TA-33BB-O, TA33BB-CMS, NK-219mm-O, NK-219mm-O x TA-33BB-O, and TA-33BB-CMS x NK-219mm-O. (PDF 554 kb) [file 41065_2016_15_MOESM2_ESM.pdf]

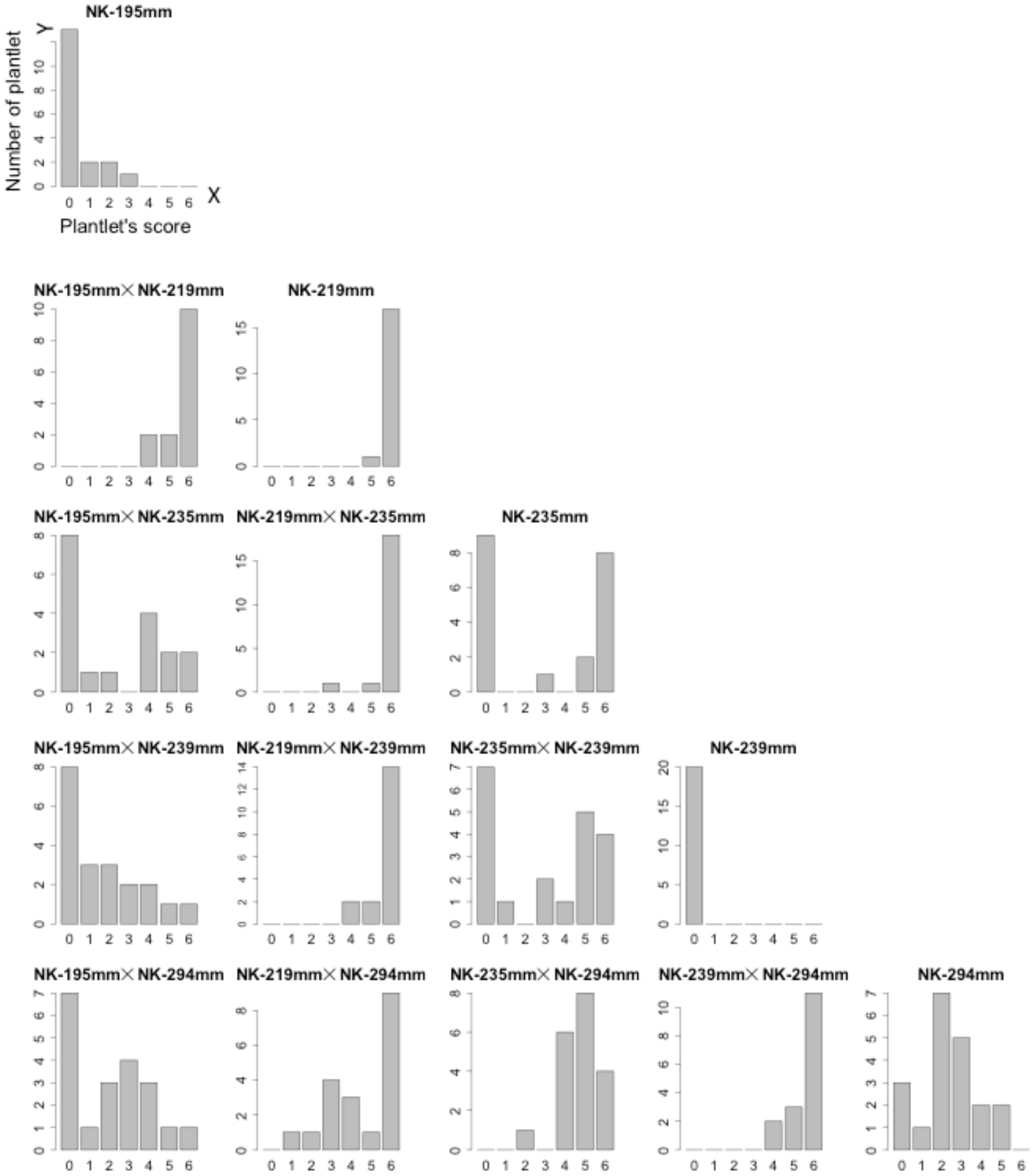

Figure S1. Distribution of callus formation frequencies in five sugar beet lines and ten F_1 populations. Suffixes are omitted. The distribution of callus formation frequencies in F_1 populations and their parental lines are shown. For this analysis, each plant received a score of either 0, 1, 2, 3, 4, 5, or 6. X-axes are the score. Y-axes are number of plants. NK-195mm-O, NK-219mm-O, and NK-239mm-O had single-peak distributions, whereas the peak distribution for NK-294mm-O was less clear. NK-235mm-O had a bimodal distribution. In the F_1 populations, single peaks were observed except for NK-195mm-CMS x NK-294mm-O (a less clear peak), NK-195mm-CMS x NK-235mm-O, and NK-235mm-CMS x NK-239mm-O (bimodal distributions). For the mean values of callus formation frequencies in the F_1 populations and parental lines, see Table 1.

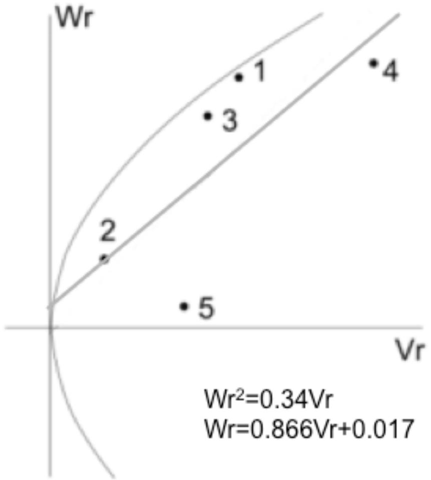

Fig. S2. A (Vr, Wr) plot for callus formation. Parental array points are NK-195mm-O (1), NK-219mm-O (2), NK-235mm-O (3), NK-239mm-O (4), and NK-294mm-O (5).

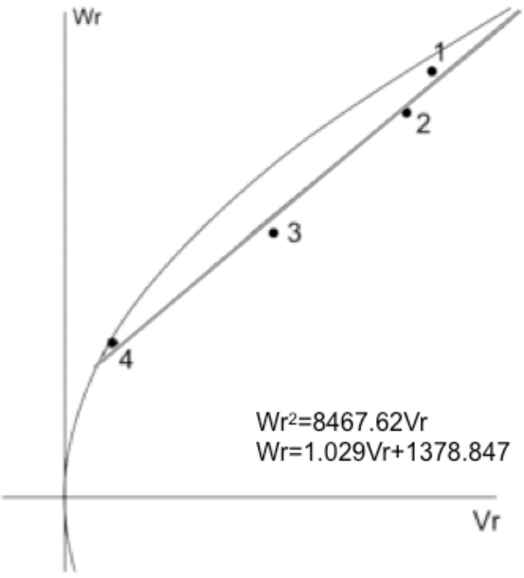

Fig. S3 A (Vr, Wr) plot of the regeneration scores. Parental array points are NK-195mm-O (1), NK-219mm-O (2), NK-235mm-O (3), and NK-294mm-O (4).

Haplotype analysis of the B *gene*

Total cellular RNA was isolated from green leaves using an RNeasy Plant Mini Kit (Qiagen,Valencia, CA). RNA samples were treated with RNase-free DNase I and reverse transcribed with SuperScript III FirstStrand Synthesis System for RT-PCR (Life Technologies, Carlsbad, CA) using oligo dT primer. The resultant cDNA was mixed with PrimeSTAR Max Premix (Takara Bio, Shiga, Japan), and subjected to PCR amplification with a pair of primers designed based on EMBL/DDBJ/GenBank accession no. HQ709093 (Fig. S4). The PCR protocol was 1 x 98˚C for 1 min, 35 x (98˚C for 5 sec, 55˚C for 15 sec, and 72˚C for 1 min), and 1 x 72˚C for 3 min. The PCR products were cloned into the pBluescript (SK) plasmid using Ligation high Ver. 2 (TOYOBO, Osaka, Japan). Cloned amplicons were sequenced using an ABI3130 sequencer (Applied Biosystems, Foster City, CA).

We obtained PCR products from TA-33BB-O and NK-219mm-O. As expected, nucleotide sequences of the PCR products corresponded to exons 8 and 9 of *BvBTC1* (Fig. S4). Comparing the sequences of TA-33BB-O- and NK-219mm-O *BvBTC1*, five single-nucleotide polymorphisms (SNPs) were found between them (Fig. S4). According to Pin et al. (2012), these SNP patterns match with haplotypes 'a' and 'd', which were exclusively found in annual and biennial beets, respectively. Thus, the annual habitat of TA-33BB-O is likely conditioned by the dominant *B* allele.

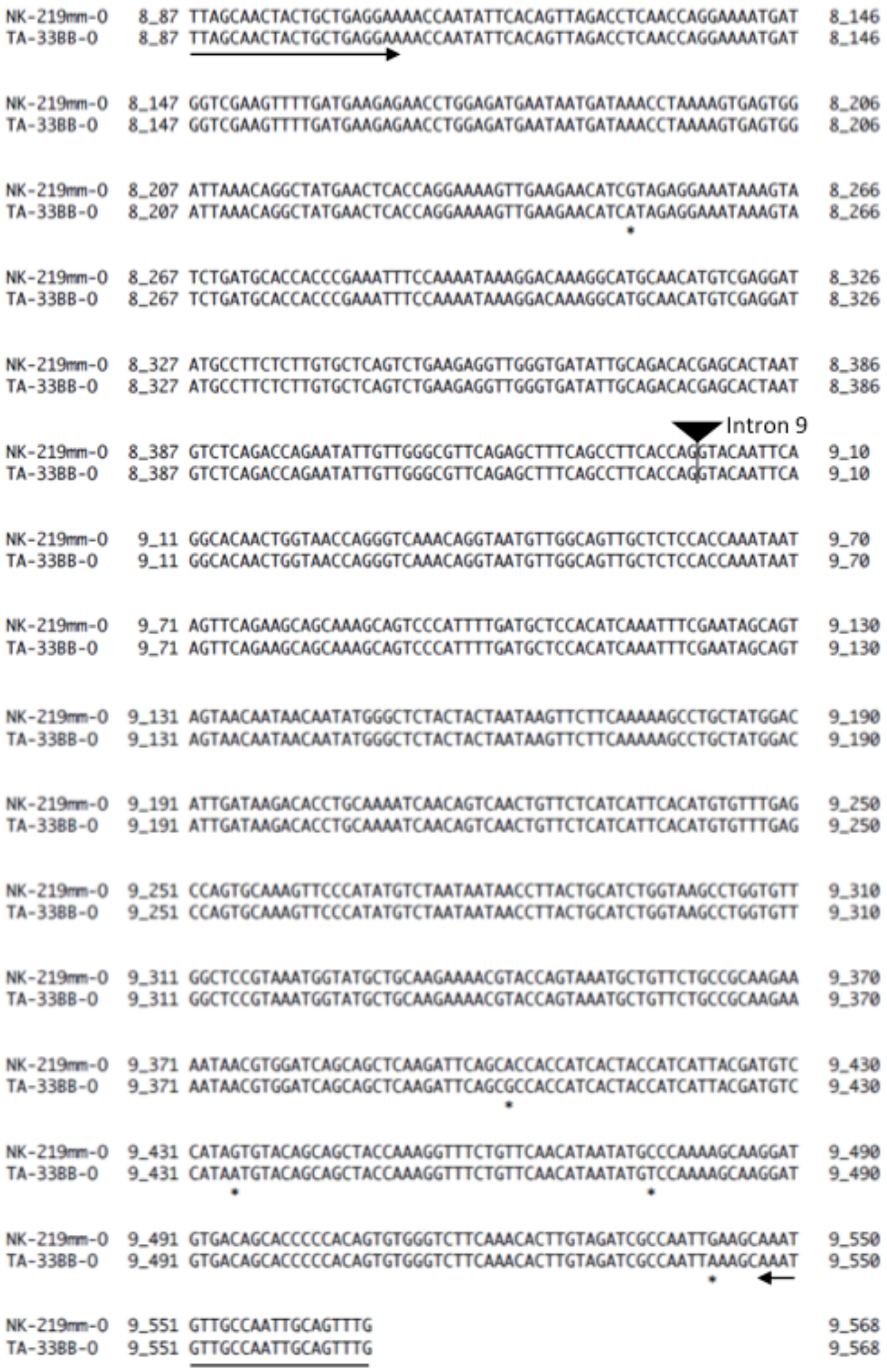

Fig. S4 Nucleotide sequences of a portion of *BvBTC1* from NK-219mm-O and TA-33BB-O are aligned. Two 918-bp sequences were PCR-amplified from leaf cDNA (see 'Haplotype analysis of the *B* gene). Nucleotide sequences are numbered from the first residue of each exon: residues are shown by exon number, under bar, and residue number. The black triangle indicates the position of an intron. Asterisks indicate nucleotide polymorphisms between NK-219mm-O and TA-33BB-O. Arrows indicate the positions of PCR primers.

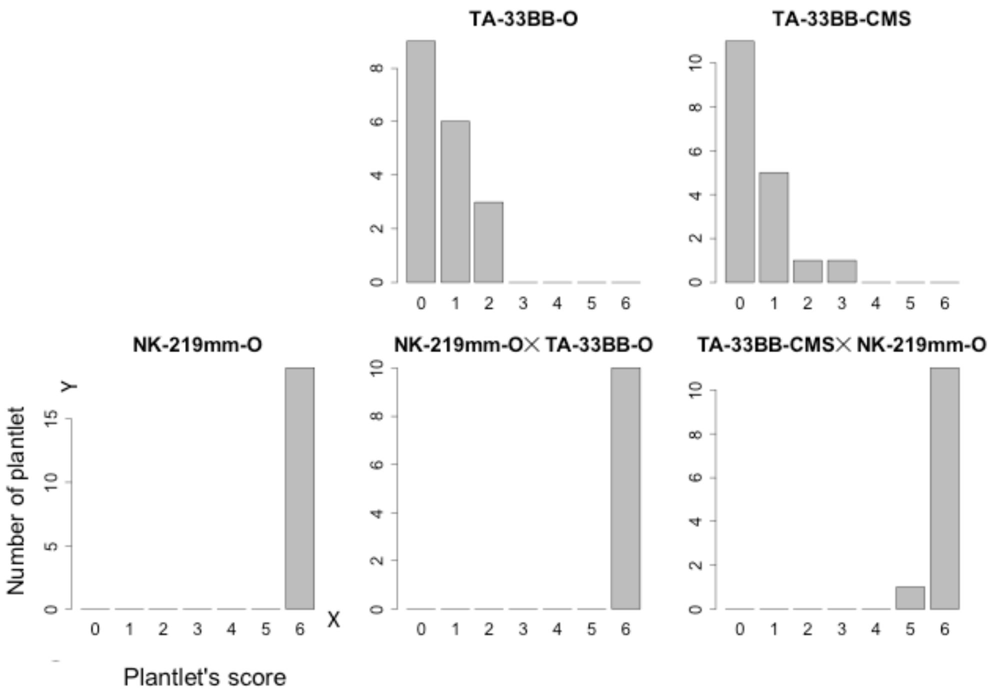

Fig. S5 The distribution of callus-formation frequencies in TA-33BB-O, TA-33BB-CMS, NK-219mm-O, NK-219mm-O x TA-33BB-O, and TA-33BB-CMS x NK-219mm-O. For this analysis, each plant received a score of either 0, 1, 2, 3, 4, 5, or 6, corresponding to the six explants used in the study. X-axes are the score. Y-axes are number of plants.

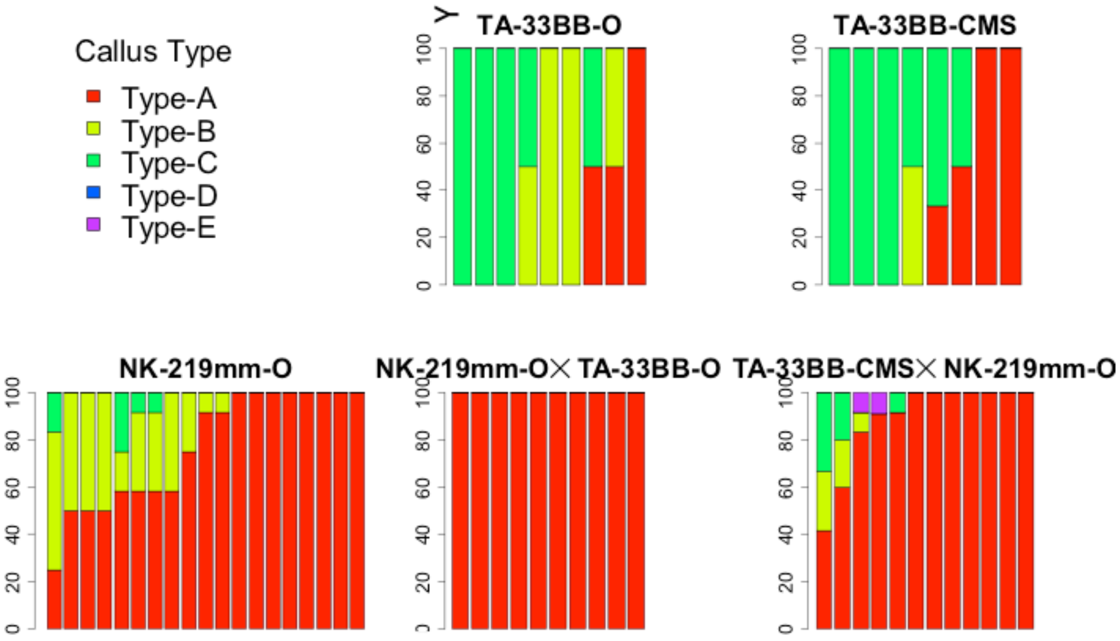

Fig. S6 Summary of callus phenotypes from TA-33BB-O, TA33BB-CMS, NK-219mm-O, NK-219mm-O x TA-33BB-O, and TA-33BB-CMS x NK-219mm-O. Each bar represents a single plant (Y-axes are the percentage of explants of each callus phenotype). The five different phenotypes are indicated by bars of different colors: red (Type-A), yellow (Type-B), green (Type-C), blue (Type-D), and purple (Type-E).

References

Pin PA, Zhang W, Vogt SH, Dally N, Büttner B, Schulze-Buxloh G, Jelly NS, Chia TY, Mutasa-Göttgens ES, Dohm JC, Himmelbauer H, Weisshaar B, Kraus J, Gielen JJ, Lommel M, Weyens G, Wahl B, Schechert A, Nilsson O, Jung C, Kraft T, Müller AE, The role of a pseudo-response regulator gene in life cycle adaptation and domestication of beet. *Current Biology*, 22: 1095-1101, 2012.
