# Supplementary material for: Efficient callus formation and plant regeneration are heritable characters in sugar beet (Beta vulgaris L.)
Source: Hereditas. 2016 Nov 15;153:12. doi: 10.1186/s41065-016-0015-z (PMC5226093; doi:10.1186/s41065-016-0015-z)
Supplement: Additional file 3: Table S1. — Absence of epistasis in callus formation, two-way ANOVA of callus formation frequencies. Table S2. Absence of epistasis in callus formation, two-way ANOVA of the unity of Wr-Vr. Table S3. Combining ability and heritability of callus formation, statistics of combining ability in callus formation. Table S4. Combining ability and heritability of callus formation, statistics of genetic variance in callus formation. Table S5. Absence of epistasis in plant regeneration, two-way ANOVA of the regeneration score. Table S6. Absence of epistasis in plant regeneration, two-way ANOVA of the unity of Wr-Vr. Table S7. Combining ability and heritability of plant regeneration, statistics of combining ability in plant regeneration. Table S8. Combining ability and heritability of plant regeneration, statistics of combining ability in plant regeneration. Table S9. In vitro performances of TA-33BB-O, TA-33BB-CMS, NK-219mm-O, and their F1 populations. (PDF 110 kb) [file 41065_2016_15_MOESM3_ESM.pdf]

Tables S1 and S2. *Absence of epistasis in callus formation*

On the basis of data presented in Table 1, quantitative genetic analyses were possible. Values in Table 1 were arcsine-converted and further analyzed. The results of a two-way ANOVA are shown in Table S1. Whereas differences between replications were non-significant, differences among genotypes were significant at the 1% level. Using DIAL98, a program for diallel analysis (Ukai 1989), a plot was constructed (Fig. S2), in which array variances and co-variances with the non-recurrent parents (Vr and Wr, respectively) in the diallel were plotted (Hayman 1954). In this plot, the regression coefficient was 0.866, suggesting the absence of epistasis, which would be non-negligible if the value was significantly less than 1. This notion was supported by a two-way ANOVA for the unity of Wr-Vr (Table S2), where the differences in array and block design were insignificant.

Table S1. Two-way ANOVA of frequencies of callus formation

Source	Degrees of freedom（DF）	Sum of squares（SS）	Mean of squares（MS）	F
Replication	1	0.05	0.05	2.88[ns]
Genotype	14	6.65	0.47	28.71[**]
Error	14	0.23	0.02	
Total	29			

[ns]Non-significant; [**]Significant at the 1% level.

Table S2. Two-way ANOVA of the unity of Wr-Vr

Source	Degrees of freedom（DF）	Sum of squares（SS）	Mean of squares（MS）	F
Array difference	4	0.039	0.010	4.91[ns]
Block difference	1	0.001	0.000	0.32[ns]
Error	4	0.008	0.002	

[ns]Non-significant.

Tables S3 and S4. *Combining ability and heritability of callus formation*.

Using the method of Griffing (1956), we calculated the combining ability for callus formation and found that both general combining ability (GCA) and specific combining ability (SCA) were significant (p<0.01) (Table S3). The statistics for the genetic variance of callus formation is shown in Table S4. Values for both broad-sense and narrow-sense heritability are high.

Table S3 Combining ability in callus formation

Variation	Degrees of freedom（DF）	Sum of squares (SS)	Mean of squares (MS)	F
Replication	1	0.03	0.03	1.47^{ns}
General Combining Ability	4	2.11	0.53	22.56^{**}
Specific Combining Ability	5	1.04	0.21	8.85^{**}
Error	9	0.21	0.02	45.36^{**}
Total	19	3.39	0.08	5.03^{*}

^{ns}Non-significant; **Significant at 1% level; *Significant at 5% level.

Table S4 Statistics of genetic variance in callus formation

Genetic parameters and statistics	
D (additive variance)	0.3351
H_1 (dominance variance 1)	0.3538
H_2 (dominance variance 2)	0.3253
$\sqrt{H_1/D}$ (average degree of dominance)	1.028
$H_2/4H_1$ (proportion of dominant genes)	0.5307
h^2/H_2 (number of genes)	1.0802
h (average direction of dominance)	0.5352
$h_{(ns)}$ (heritability for diallel in a broad sense)	0.963
$h_{(bs)}$ (heritability for diallel in a narrow sense)	0.64

Tables S5 and S6. *Absence of epistasis in plant regeneration*

We performed a two-way ANOVA using the regeneration score from Table 1. The results are shown in Table S5. Differences between replicates were insignificant, but differences between genotypes were significant at the 1% level. DIAL98 was used to draw a (Vr, Wr) plot (Fig. S3). In this plot, the regression coefficient was 1.029, suggesting the absence of epistasis. This notion was supported by a two-way ANOVA for the unity of Wr-Vr (Table S6), where the differences in array and in block design were both insignificant.

Table S5. Two-way ANOVA of the regeneration score

Source	Degrees of freedom（DF）	Sum of squares（SS）	Mean of squares（MS）	F
Replication	1	598.3872	598.39	5.12^{ns}
Genotype	9	76740.67	8526.74	3.18^{**}
Error	9	3116.055	346.23	
Total	19			

^{ns}Non-significant; **Significant at 1% level.

Table S6 Two-way ANOVA of the unity of Wr-Vr

Source	Degrees of freedom（DF）	Sum of squares（SS）	Mean of squares（MS）	F
Array difference	3	213398.5	71132.82	0.
Block difference	1	23082.52	23082.52	0.
Error	3	569152.5	189717.5	

^{ns}Non-significant.

Tables S7 and S8. *Combining ability and heritability of plant regeneration*

The GCA and SCA for plant regeneration are shown in Table S7. The statistics of genetic variance in plant regeneration is shown in Table S8.

Table S7 Statistics of combining ability in plant regeneration

Variation	Degree of freedom (DF)	Sum of square (SS)	Mean of square (MS)	F
Replication	1	54.19	54.19	2.12
General Combining Ability	3	20670.77	6890.26	268.98**
Specific Combining Ability	2	6342.87	3171.44	123.80**
Error	5	128.08	25.62	7.16*
Total	11	27195.91	254.61	0.77

nsNon-significant; **Significant at 1% level; *Significant at 5% level.

Table S8. Statistics of genetic variance in plant regeneration

Genetic parameters and statistics	
D (additive variance)	8233.843
H_1 (dominance variance 1)	2626.827
H_2 (dominance variance 2)	2598.687
$\sqrt{H_1/D}$ (average degree of dominance)	0.565
$H_2/4H_1$ (proportion of dominant genes)	0.5435
h^2/H_2 (number of genes)	0.5357
h (average direction of dominance)	-33.3881
$h_{(ns)}$ (heritability for diallel in a broad sense)	0.976
$h_{(bs)}$ (heritability for diallel in a narrow sense)	0.831

Callus formation and potential of plant regeneration in annual and biennial beets

Seeds of TA-33BB-O, TA-33BB-CMS, NK-219mm-O, NK-219mm-O x TA-33BB-O, and TA-33BB-CMS x NK-219mm-O were surface sterilized and sown to obtain plantlets for *in vitro* culture (for the procedure and phenotypic evaluation, see Findings and references therein). In Table S9, the frequencies of callus formation and regeneration scores are shown. See Findings for description.

Table S9 *In vitro* performance of TA-33BB-O, TA-33BB-CMS, NK-219mm-O, and their F_1 populations

	TA-33BB-O	TA-33BB-CMS	NK-219mm-O	NK-219mm-O x TA-33BB-O	TA-33BB-CMS x NK-219mm-O
Frequency of callus formation	11.11±12.78	10.19±14.16	100.00±0.00	100.00±0.00	98.61±4.81
Regeneration score	77.78±71.20	77.08±84.02	174.12±28.99	200.00±0.00	182.37±29.4

References

Griffing B, Concept of general and specific combining ability in relation to diallel crossing systems. *Australian Journal of Biological Sciences*, 9: 463-493, 1956.

Hayman BI, The theory and analysis of diallel crosses. *Genetics*, 39: 789-809, 1954.

Ukai Y, A microcomputer program DIALL for diallel analysis of quantitative characters. *Japanese Journal of Breeding* 39: 107-109, 1989; https://web.archive.org/web/20070802060224/http://lbm.ab.a.u-tokyo.ac.jp/~ukai/dial98.html (accessed on 4 November 2016)
